# Generating and Expanding Conventional Type 1 Dendritic Cells

**DOI:** 10.1002/eji.70307

**Published:** 2026-10-28

**Authors:** Sukriti Sharma, Shandré Pillay, Christian T. Mayer

**Affiliations:** ^1^ Experimental Immunology Branch, Center for Cancer Research, National Cancer Institute National Institutes of Health Bethesda Maryland USA

**Keywords:** cDC1, cellular engineering, dendritic cell differentiation, hematopoietic progenitors, immunotherapy

## Abstract

Conventional type 1 dendritic cells (cDC1s) are specialized antigen‐presenting cells that play essential roles in cross‐presentation, immunity to intracellular pathogens, and antitumor immune responses. Their low abundance and limited accessibility in blood and many tissues have driven extensive efforts to generate and expand cDC1s for mechanistic and translational studies. Over the past two decades, advances in cDC1 biology have transformed the field from heterogeneous bone marrow‐derived cultures to increasingly sophisticated platforms that generate, enrich, or expand bona fide cDC1s, alongside approaches whose products remain best described as cDC1‐like. Here, we review cytokine‐driven cultures, Notch ligand‐supported systems, human CD34^+^ progenitor and induced pluripotent stem cell‐derived platforms, transcription factor‐based programming, and endogenous cDC1 expansion. We distinguish bona fide cDC1s, cDC1‐enriched cultures, and cDC1‐like cells and compare these approaches by lineage validation, enrichment, yield, culture duration, function, scalability, and translational maturity. Together, these platforms provide complementary tools for cDC1 research and therapeutic development.

## Graphical abstract

Conventional type 1 dendritic cells (cDC1s) are difficult to obtain in sufficient numbers. This Review compares strategies based on progenitor differentiation, cell programming, or expansion in tissues, and shows how differences in cell identity, population composition, function, and yield should guide platform selection.

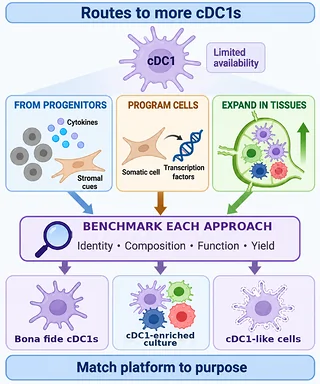

**Abbreviations**
CCR7: C–C motif chemokine receptor 7
cDC: conventional dendritic cell
cDC1: conventional type 1 dendritic cell
cDC2: conventional type 2 dendritic cell
CDP: common dendritic cell progenitor
DC: dendritic cell
DLL1: Delta‐like 1
Flt3L: Fms‐like tyrosine kinase 3 ligand
GM‐CSF: granulocyte‐macrophage colony‐stimulating factor
GMP: good manufacturing practice
HSC: hematopoietic stem cell
iCD103: induced CD103^+^ dendritic cell
iDC1: induced type 1 conventional dendritic cell
IL‐4: interleukin‐4
iPSC: induced pluripotent stem cell
MPP: multipotent progenitor
pDC: plasmacytoid dendritic cell
SCF: stem cell factor
TLR: Toll‐like receptor

## 1 Introduction

Conventional type 1 dendritic cells (cDC1s) are a specialized dendritic cell (DC) subset that plays central roles in cross‐presentation, immunity to intracellular pathogens, and antitumor immune responses [1, 2]. Through their ability to efficiently process and present cell‐associated antigens and coordinate adaptive and innate immune responses, cDC1s are critical regulators of protective immunity in both mice and humans [3, 4]. Their importance has been demonstrated in diverse settings including viral and parasitic infections, cancer, vaccination, and immune tolerance [1, 5].

Over the past decade, major advances have been made in defining the developmental pathways, transcriptional networks, and functional specialization of cDC1s. Recent reviews have focused on cDC1 biology, ontogeny, maturation, function, and therapeutic exploitation [6, 7, 8, 9, 10, 11], whereas comparatively less attention has been devoted to critically comparing platforms for generating and expanding cDC1s. This gap has become increasingly important as cDC1s are now widely used not only for mechanistic studies but also as platforms for cellular engineering and immunotherapy [6, 12].

The difficulty of obtaining sufficient cDC1s from blood and tissues has long represented a major experimental and translational challenge. Consequently, considerable effort has been devoted to developing experimental systems that generate cDC1s ex vivo for mechanistic studies and therapeutic applications. Over the past two decades, these approaches have evolved from empirical cytokine‐driven cultures to increasingly sophisticated platforms informed by advances in cDC1 biology. Rather than relying on a single strategy, the field has developed complementary approaches that expand progenitor populations, promote cDC1 differentiation and maintenance, provide developmental niche signals, induce cDC1‐associated programs in other cell types, or expand endogenous cDC1s.

As a result, the field now encompasses a diverse range of approaches, including cytokine‐driven differentiation systems, Notch ligand‐supported cultures, human CD34^+^ progenitor‐based platforms, induced pluripotent stem cell (iPSC)‐derived methods, transcription factor‐based programming strategies, and approaches to expand endogenous cDC1s in vivo. The major experimental platforms are summarized in Figure 1, their underlying biological principles are illustrated in Figure 2, and their relative strengths and limitations are compared in Table 1. This Review examines how advances in cDC1 biology have driven complementary strategies for generating and expanding cDC1s and evaluates the evidence supporting lineage identity, the degree of correspondence to relevant primary cDC1 states, scalability, and translational use across current platforms.

**FIGURE 1:**
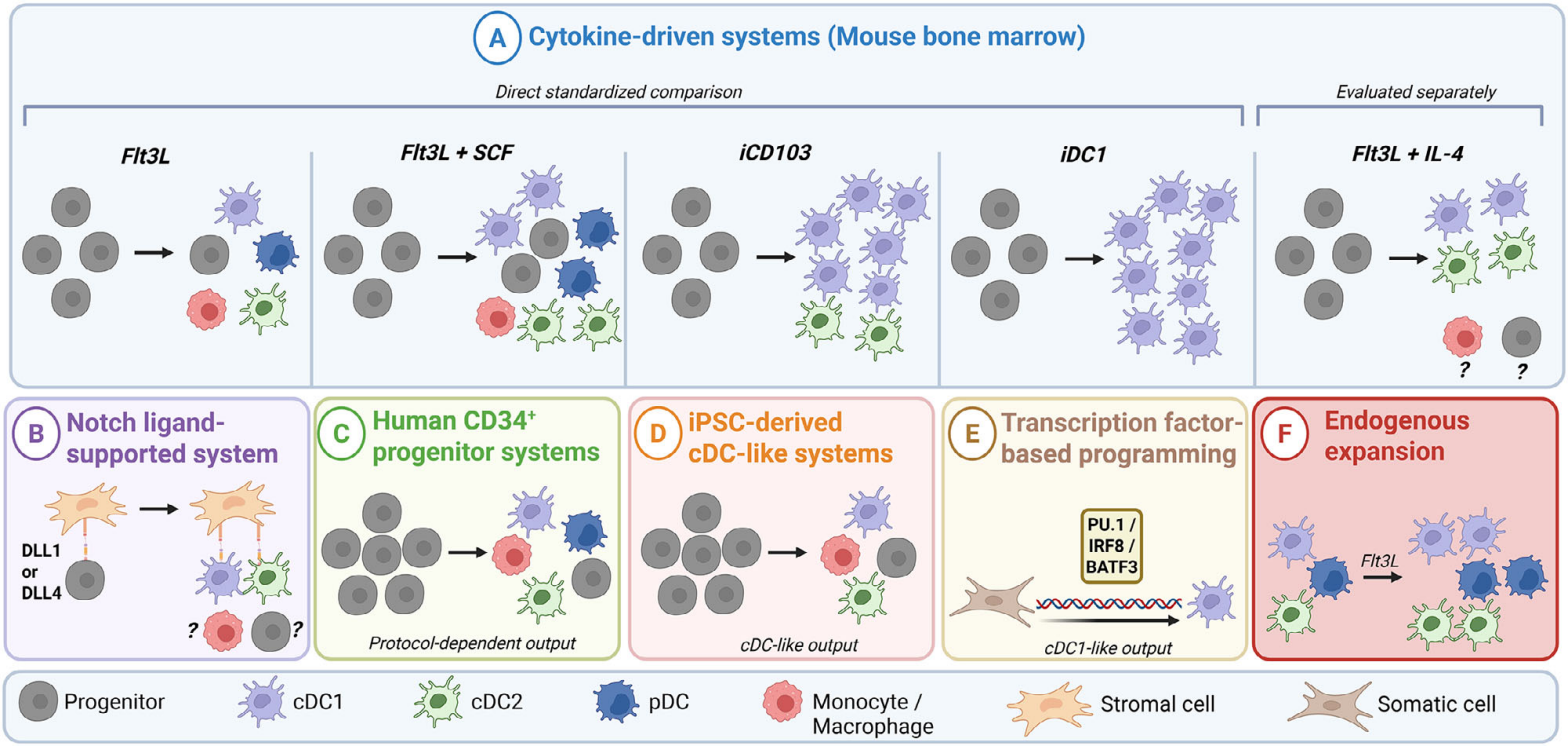
Experimental platforms for generating and expanding cDC1s. Schematic overview of ex vivo and in vivo approaches used to generate, enrich, or expand cDC1s or cDC1‐like cells. (A) Cytokine‐driven mouse bone marrow cultures. Flt3L, Flt3L+SCF, iCD103, and iDC1 were compared directly under standardized conditions, whereas Flt3L+IL‐4 was evaluated separately. Flt3L and Flt3L+SCF generate heterogeneous cultures; iCD103 enriches bona fide cDC1s while retaining a residual cDC2 population; and iDC1 generates high‐purity bona fide cDC1s. In whole bone marrow cultures, Flt3L+IL‐4 increases cDC1 and cDC2 output while suppressing pDC development; macrophage‐like cells and residual progenitors were not assessed and are shown with question marks. (B) Notch ligand‐supported cultures generate cDC1s and cDC2s, whereas macrophage‐like, progenitor, and other non‐DC fractions are not consistently reported. (C) Human CD34^+^ progenitor systems generate protocol‐dependent mixtures of cDC1 or cDC1‐like cells and other myeloid populations. (D) iPSC‐derived systems generate heterogeneous cDC‐like and monocyte‐like populations. (E) Transcription factor‐based programming induces cDC1‐like states directly from somatic cells. (F) Flt3L‐mediated endogenous expansion increases multiple Flt3‐responsive DC populations, including cDC1s. The brackets in Panel A distinguish platforms compared directly under standardized conditions from a platform evaluated separately. Icon numbers and relative frequencies are schematic. Question marks denote populations not assessed in the IL‐4 study or not consistently quantified in Notch‐supported systems. In Panels C–E, the cDC1‐shaped icon denotes protocol‐dependent cDC1 or cDC1‐like output as indicated by the panel labels and does not imply equivalent developmental validation. Outputs differ in purity, culture composition, developmental state, and strength of lineage validation. DLL1, Delta‐like 1; DLL4, Delta‐like 4. Created in BioRender. Mayer, C. (2026) https://BioRender.com/41m02hj.

**FIGURE 2:**
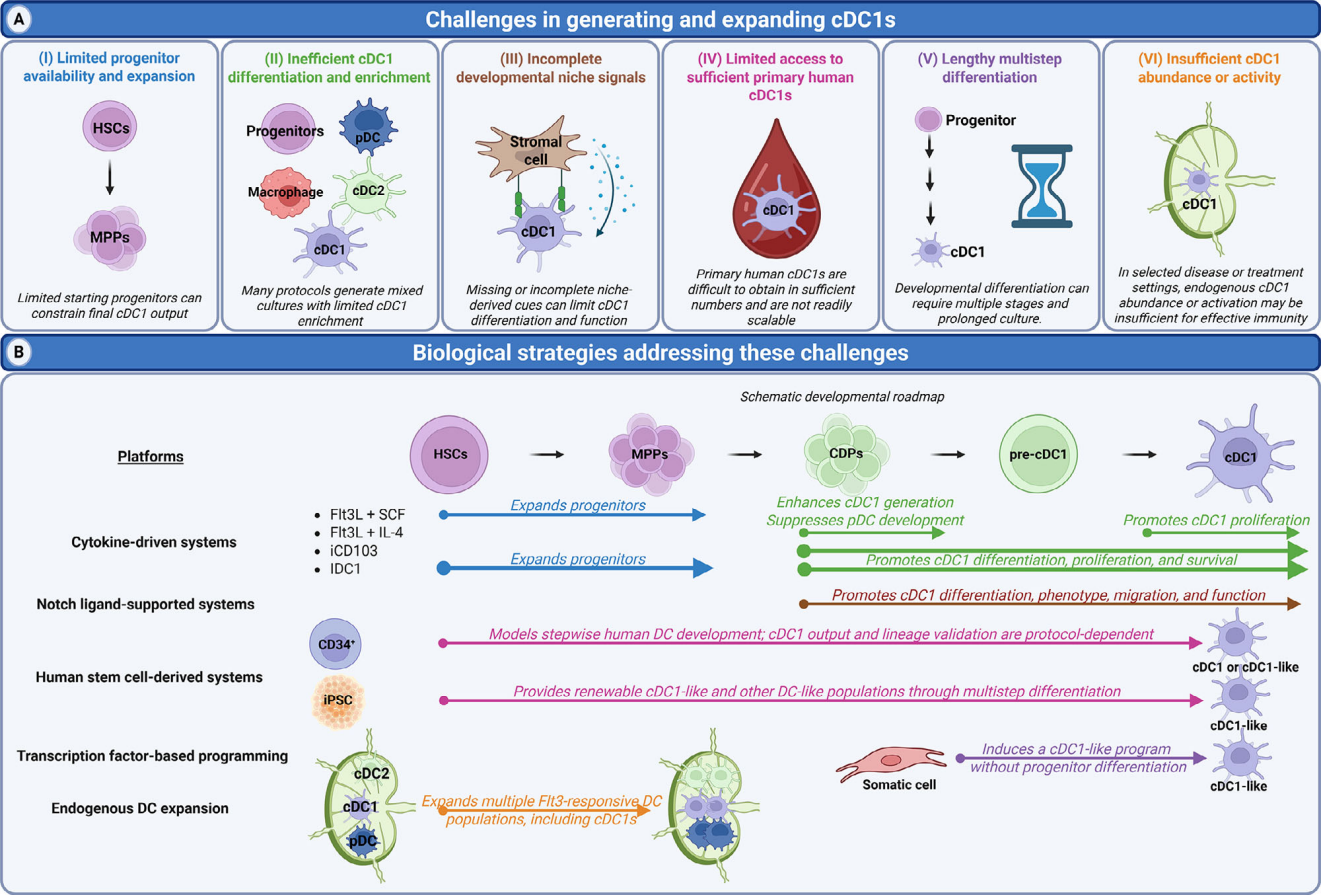
Biological challenges and strategies in cDC1 generation and expansion. (A) Major biological and technical challenges include limited progenitor availability and expansion, inefficient cDC1 differentiation and enrichment, incomplete developmental niche signals, limited access to sufficient primary human cDC1s, lengthy multistep differentiation, and insufficient cDC1 abundance or activity in selected disease or treatment settings. Developmental niche signals are depicted schematically and may include both membrane‐bound and soluble cues. (B) Conceptual overview of how current strategies address distinct stages and bottlenecks in cDC1 biology. Flt3L+SCF expands upstream progenitors; Flt3L+IL‐4 enhances cDC1 generation from early DC progenitors, promotes cDC1 proliferation, and suppresses pDC development; iCD103 and iDC1 share an Flt3L plus low‐dose GM‐CSF phase that promotes cDC1 differentiation, proliferation, and survival, whereas iDC1 additionally incorporates SCF‐mediated progenitor expansion. Notch ligand‐supported systems provide membrane‐bound signals that promote cDC1 differentiation, phenotype, migration, and function, although the relevant developmental stage and quantitative requirement depend on species and progenitor context. Human CD34^+^ progenitor systems model stepwise human DC development and generate cDC1 or cDC1‐like populations with protocol‐dependent output and lineage validation, whereas iPSC‐derived systems provide renewable cDC1‐like and other DC‐like populations through multistep differentiation. Transcription factor‐based programming induces a cDC1‐like program without recapitulating progenitor differentiation. Endogenous Flt3L administration expands multiple Flt3‐responsive DC populations, including cDC1s. The developmental roadmap integrates mouse and human concepts. Arrows indicate principal reported effects and do not imply exclusive action at a single developmental stage. The Flt3L+IL‐4 arrow represents its progenitor‐level mechanism and does not imply selective cDC1 output in whole bone marrow cultures. CDP, common dendritic cell progenitor; HSC, hematopoietic stem cell; MPP, multipotent progenitor; pre‐cDC1, cDC1‐committed precursor. Created in BioRender. Mayer, C. (2026), https://BioRender.com/rt3z0wn.

**TABLE 1:** Choosing the appropriate cDC1 platform.

Platform	Species, starting material, and duration	Reported cDC1 output	Identity and functional benchmarking	Principal strengths	Principal limitations/maturity	Best‐supported use; references
**Mouse cytokine‐driven systems**						
Flt3L culture	Mouse bone marrow; 9 days	15.2% of total live cells (9.1% CD103^+^ and 6.1% CD103^−^ cDC1); also cDC2, pDC, macrophage‐like, and other cells	CD24/XCR1 phenotype; CD103^+^ and CD103‐ populations align transcriptionally with primary cDC1; cross‐presentation established	Simple, rapid, genetically tractable; generates several DC subsets	Low cDC1 selectivity; substantial whole‐culture heterogeneity	DC development and subset comparisons; [14, 23, 24]
Flt3L+SCF	Mouse bone marrow; 11 days	Higher cDC1 frequency and absolute output than Flt3L alone; mixed Flt3‐responsive output retained	Phenotypically defined cDC1; standardized whole‐culture comparison; no separate RNA‐seq of this condition	Recruits and expands HSCs and early progenitors; increases total DC output	Not cDC1‐selective; mixed culture	Progenitor expansion and higher‐output mixed DC cultures; [14, 28]
Flt3L+IL‐4	Mouse bone marrow; 8 days	cDC1 and cDC2 absolute output increased; pDC development suppressed; 39% cDC1 and 61% cDC2 within the DC compartment; total‐culture purity not reported	Phenotypic, transcriptional, and functional characterization; early IL‐4Rα‐STAT6‐dependent effect and increased cDC1 proliferation	Rapid, feeder‐free; defines progenitor‐level IL‐4 effects	Mixed cDC1/cDC2 output; macrophage‐like, progenitor, and other non‐DC fractions not assessed; indirect comparison	DC‐progenitor fate and cytokine signaling; [29]
iCD103	Mouse bone marrow; 16 days	67.2% CD103^+^ cDC1 and 24.7% cDC2 of total live cells; pDC, macrophage‐like, and other fractions each ≤3.3%	Batf3 dependence; cDC1 phenotype and transcriptional alignment; innate responses and cross‐presentation	Enriches bona fide CD103^+^ cDC1	Residual cDC2 population; longer culture; lower output than iDC1	Enriched mouse cDC1 for mechanistic and functional studies; [14, 19]
iDC1	Mouse bone marrow; 23 days	92.8% CD103^+^ cDC1 in the direct comparison and >95% in optimized cultures; about 1.5 × 10^9^ cDC1 per mouse; ≥75‐fold more than the original recombinant‐cytokine iCD103 method	XCR1/CLEC9A/CD24; +32‐kb Irf8 enhancer dependence; RNA‐seq and ImmGen alignment; innate responses and cell‐associated antigen cross‐presentation	Highest purity and yield among directly compared mouse cytokine systems; scalable	Longest culture; serial replating	High‐purity bona fide mouse cDC1 generation and cellular engineering; [14]
**Notch‐supported systems**						
Mouse Notch ligand‐supported culture	Mouse bone marrow or HoxB8‐derived progenitors; about 7 days	Mixed cDC1/cDC2 culture; standardized total‐live cDC1 purity and other non‐DC output not reported	CD8α/CD103/DEC205/CCR7 phenotype; transcriptional similarity to splenic cDC1; migration, cross‐priming, and antitumor vaccination	Promotes terminal differentiation and splenic cDC1‐associated function	Feeder‐dependent, multilineage, and incompletely characterized at the whole‐culture level	Notch biology and developmental‐niche studies; [13, 38]
**Human CD34^+^ progenitor systems**						
Early cytokine‐based or engineered‐niche systems	Human cord blood, mobilized or nonmobilized blood, or bone marrow CD34^+^ progenitors; about 18–21 days	cDC1 generally remain a minority within heterogeneous cultures; outputs and denominators vary by protocol	XCR1/CD141/CLEC9A phenotype; selected protocols include transcriptional comparison, innate responsiveness, and cross‐presentation	Models human DC development; includes feeder‐free and engineered‐niche variants	Variable output, composition, and strength of lineage validation	Human DC ontogeny and mechanistic studies; [15, 42, 43, 44]
Human Notch‐supported developmental cultures	Human bone marrow progenitors: 14 days; cord‐blood CD34^+^ progenitors: 7‐day expansion plus 14–21‐day differentiation	DLL1 markedly increases cDC1 output, but cultures remain multilineage; complete whole‐culture composition is not consistently reported	IRF8 dependence; phenotype, TLR responses, cytokine production, and single‐cell transcriptional benchmarking	Strong mechanistic platform for human Notch‐dependent DC differentiation	Feeder‐dependent, relatively long, and heterogeneous	Human cDC1 developmental and niche‐signal studies; [13, 39]
Feeder‐free Flt3L/GM‐CSF cDC1‐like protocol	Human mobilized peripheral‐blood CD34^+^ progenitors; about 14 days	Large CD141^+^CLEC9A^+^ output reported; standardized total‐culture purity and a common yield denominator not reported	Primarily phenotypic characterization; comprehensive developmental, transcriptomic, and functional benchmarking against primary cDC1s not reported	Feeder‐free and comparatively simple	Best classified as cDC1‐like; incomplete lineage validation	Human cDC1‐like cell generation and protocol development; [45]
Optimized serum‐free DLL1‐supported platform	Human cord blood, mobilized or nonmobilized blood, or bone marrow CD34^+^ progenitors; 7‐day expansion plus 12‐day differentiation	>40% cDC1 in DLL1 cultures; up to about 2,900 cDC1 per input cord‐blood HSC, 436 per mobilized‐blood HSC, and 404 per bone‐marrow HSC	CLEC9A/CADM1/XCR1/CD141; bulk transcriptomics; cell‐associated antigen cross‐presentation and preclinical vaccination	Very high output from several human progenitor sources; serum‐free	Feeder‐dependent; preclinical rather than clinically validated	Large‐scale preclinical human cDC1 production and vaccine development; [46]
Feeder‐free GMP‐compatible CD34^+^ platform	Human cord‐blood CD34^+^ HSPCs; 14 days, then purification and maturation	Mean 705‐fold and up to 1614‐fold expansion; about 1–8 × 10^9^ purified cDC1 from 5 × 10^6^ input cells (mean about 3.5 × 10^9^)	Phenotypic and single‐cell transcriptional benchmarking; cross‐presentation, cytokine production, and humanized‐mouse antitumor activity	Feeder‐free, GMP‐compatible, and very high reported output	Includes purification; preprint; clinical readiness not established	Emerging preclinical manufacturing platform; [47], preprint
**Renewable, programmed, and endogenous approaches**						
iPSC‐derived cDC1‐like systems	Human iPSCs; typically 3–5 weeks	Heterogeneous cDC‐like output; no common standardized cDC1 purity or yield denominator	cDC1‐associated phenotype and cross‐presentation in early studies; single‐cell similarity to blood cDC1 in one protocol; other outputs closer to monocyte‐derived DCs	Renewable and engineerable starting material	Prolonged multistep differentiation; mixed output; protocol‐dependent correspondence; preclinical	Disease modeling and cellular engineering; [48, 49, 50, 51]
Transcription factor‐based programming	Mouse or human fibroblasts, mesenchymal stromal cells, or tumor cells; about 9 days ex vivo; in vivo regimens vary	Study‐specific marker‐defined conversion; no standardized bona fide cDC1 purity or yield denominator	Global cDC1‐like transcriptional program; PU.1/IRF8/BATF3 chromatin engagement; antigen uptake, cytokine production, and cross‐presentation	Rapid, feeder‐free, and bypasses progenitor differentiation	No expandable intermediate; delivery, safety, and stability remain unresolved; preclinical	Mechanistic reprogramming and preclinical cellular or in situ immunotherapy; [52, 53, 54]
Endogenous DC expansion	Mouse or human, in vivo; treatment‐ and regimen‐dependent	No ex vivo purity or yield metric; Flt3L expands several Flt3‐responsive DC populations, including cDC1	cDC1 contribution shown in defined combination regimens, including Batf3‐deficient bone‐marrow chimeras; dependence is context‐specific	Preserves physiological antigen acquisition and tissue localization	Limited control over subset identity, dose, and activation state; usually requires combination treatment	In vivo DC expansion and combination immunotherapy; [20, 21, 55, 56, 57, 58]

*Note*: Values are reported as described in the cited studies and are not directly comparable unless explicitly stated. Percentages refer to total live culture output unless otherwise indicated. Values for mouse Flt3L, Flt3L+SCF, iCD103, and iDC1 derive from a standardized head‐to‐head comparison [14], whereas Flt3L+IL‐4 was evaluated separately [29]; its percentages refer to the DC compartment, and macrophage‐like, residual progenitor, and other non‐DC fractions were not assessed. Human yields use the denominator reported in each study and vary with progenitor source, feeder support, cytokine composition, and endpoint. “Bona fide cDC1” and “cDC1‐like” are used according to the definitions in the main text. References are plain‐text cross‐references to the manuscript bibliography; preprints are identified explicitly.

## 2 Defining and Benchmarking Generated cDC1s

Comparisons among cDC1‐generation platforms require explicit separation of cell identity from culture composition and from correspondence to any particular tissue state [2, 8]. No single surface marker or function establishes cDC1 identity [1, 2, 8]. In mice, CD8α and CD103 vary with tissue and differentiation state, and CD11b can be low or variable rather than uniformly absent [1, 8, 13, 14]. In humans, CD141 likewise supports but does not independently establish cDC1 identity [4, 8, 15]. Cross‐presentation and cytokine production are also not lineage‐specific [1, 2, 8]. Confidence in cDC1 assignment is strongest when multiple independent lines of evidence converge, including a cDC1‐associated phenotype such as XCR1 and CLEC9A expression [4, 8, 15], absence of a dominant cDC2, monocyte, or macrophage program [14, 15]; consistency with cDC1 developmental circuitry such as the IRF8–BATF axis [7, 11]; transcriptional alignment with appropriate primary cDC1 reference populations [14, 15]; and characteristic functional properties [1, 2, 8, 14, 15].

We use “bona fide cDC1s” to denote assignment to the cDC1 lineage, not identity to any single tissue cDC1 state. Primary cDC1s are themselves heterogeneous across tissues, developmental stages, and activation states [8], and in vitro‐generated cells are expected to retain culture‐dependent features [13, 14, 15]. We use “cDC1‐like cells” for cells that display selected cDC1‐associated features but remain incompletely validated with respect to lineage identity or developmental origin. Some studies use this designation because cells lack CD8α or CD103 or retain low or variable CD11b [13]; under our framework, those features alone are insufficient because their expression varies with tissue and state. The term “cDC1‐enriched culture” describes culture composition rather than cell identity: a mixed culture can be enriched for bona fide cDC1s, whereas a highly homogeneous population with incomplete lineage validation may still consist of cDC1‐like cells. These designations reflect the totality of available evidence, recognizing that published studies differ in the types and depth of validation performed.

In this Review, “generation” or “differentiation” denotes production through a developmental pathway, “expansion” denotes an increase in cell number, “enrichment” denotes an increase in relative frequency, “mobilization” denotes increased endogenous abundance or recruitment in vivo, and “programming” or “reprogramming” denotes enforced acquisition or conversion of cell identity.

## 3 Cytokine‐Driven cDC1 Generation

The first widely adopted methods for generating DCs in vitro relied on bone marrow cultures supplemented with granulocyte‐macrophage colony‐stimulating factor (GM‐CSF) [16, 17]. These cultures provided an accessible source of DC‐like populations and became a standard experimental system for studying DC biology. However, subsequent studies demonstrated that GM‐CSF‐derived bone marrow cultures are heterogeneous and contain substantial numbers of macrophage‐like cells [14, 18]. Although these cultures remain experimentally useful, they do not faithfully model bona fide cDC1 development [18, 19].

A major advance came with the recognition that Fms‐like tyrosine kinase 3 ligand (Flt3L) is the principal cytokine regulating physiological DC development [20, 21, 22]. Unlike GM‐CSF cultures, Flt3L‐driven bone marrow cultures generate plasmacytoid DCs (pDCs) and conventional DCs (cDCs), including cDC1 and conventional type 2 DCs (cDC2s) that more closely resemble their in vivo counterparts [23, 24]. Flt3L cultures therefore became the foundation of modern cDC1‐generation strategies and remain widely used for mechanistic studies of DC development and function. Related approaches using conditionally immortalized Hoxb8‐expressing progenitors have further expanded the experimental utility of Flt3L‐based systems by providing a renewable and genetically tractable source of DC progenitors suitable for mechanistic studies and genetic manipulation [25].

CD103 expression, however, should not be equated with cDC1 lineage identity. Flt3L cultures contain both CD103^+^ and CD103^−^XCR1^+^CD24^+^ cells with cDC1‐associated transcriptional profiles [14], whereas GM‐CSF can promote CD103 expression and cross‐presentation in cDC1‐lineage cells [19, 26, 27]. CD103 therefore represents a context‐dependent phenotype associated with many nonlymphoid cDC1s rather than a lineage‐defining marker [8, 14, 26, 27]. Its acquisition alone neither establishes cDC1 lineage identity nor demonstrates terminal differentiation or a particular maturation or tissue‐localization state [8, 13, 14, 26]. This distinction is important when comparing approaches that expand upstream progenitors with those that favor the generation, enrichment, or maintenance of CD103^+^ cDC1s.

Despite this advance, cDC1s typically represent only a fraction of the total cellular output, with Flt3L cultures also containing cDC2s, pDCs, macrophages, and progenitors (Figure 1A) [14, 24]. Subsequent strategies targeted distinct bottlenecks in progenitor recruitment, DC‐subset differentiation, and CD103^+^ cDC1 enrichment and maintenance. Supplementation with stem cell factor (SCF, Kit ligand) increased recruitment and expansion of upstream hematopoietic stem and progenitor cells, resulting in increased cDC1 production together with expansion of other Flt3L‐responsive populations (Figure 1A) [28]. More recently, interleukin‐4 (IL‐4) was shown to increase the absolute output of both cDC1s and cDC2s in Flt3L bone marrow cultures while suppressing pDC development. Progenitor analyses further showed an early, cell‐intrinsic effect of IL‐4 on cDC1 generation, and transcriptomic analyses indicated that IL‐4 promotes cDC1 proliferation (Figure 1A) [29]. However, macrophage‐like cells, residual progenitors, and other non‐DC populations were not assessed in the reported whole‐culture analysis.

Recognition that GM‐CSF contributes to the homeostasis of DCs, including nonlymphoid tissue cDC1s, in vivo [30, 31, 32] prompted investigation of whether this cytokine could similarly support cDC1 development in vitro. The resulting 16‐day induced CD103^+^ DC (iCD103‐DC) protocol combined low concentrations of GM‐CSF with Flt3L to enrich functional, Batf3‐dependent CD103^+^ cDC1s and improve their recovery (Figure 1A) [19]. Most recently, induced type 1 conventional DC (iDC1) cultures combined Flt3L, SCF, and low‐dose GM‐CSF with optimized medium and a 23‐day differentiation protocol, generating CD103+ cDC1s at greater than 95% purity and substantially higher yield than earlier cytokine‐driven systems (Figure 1A) [14]. iDC1 identity was supported by XCR1, CLEC9A, and CD24 expression, dependence on the +32‐kb Irf8 enhancer, transcriptional alignment with primary and culture‐derived cDC1s, innate responsiveness, and cross‐presentation of cell‐associated antigen [14]. Together, these developmental, phenotypic, transcriptional, and functional criteria support classification of iDC1 as bona fide cDC1s under the terminology used here.

Flt3L priming followed by delayed GM‐CSF supplementation generates CD103^+^ cDC1s together with CD301b^+^ cDC2s, with CD103^+^ cDC1s arising through differentiation of pre‐cDC1s [33, 34]. The requirement for DC‐intrinsic GM‐CSF sensing for CD103 expression in vivo is tissue‐dependent, with a specific requirement in lung cDC1s [33].

Collectively, these studies illustrate how increasing understanding of cDC1 biology transformed cytokine‐driven culture systems from empirical protocols into platforms optimized for distinct biological and technical objectives (Figure 1A). Conventional Flt3L and Flt3L+SCF systems remain heterogeneous cultures containing multiple DC and non‐DC populations; Flt3L+IL‐4 increases both cDC1 and cDC2 output while suppressing pDC development; iCD103 is a cDC1‐enriched culture containing bona fide cDC1s together with a residual cDC2 population; and iDC1 is a high‐purity culture of bona fide cDC1s. Mechanistically, SCF expands upstream progenitors; IL‐4 enhances cDC1 generation from DC progenitors, promotes cDC1 proliferation, and suppresses pDC development; low‐dose GM‐CSF promotes the generation and maintenance of CD103^+^ cDC1s; and iDC1 combines progenitor expansion and low‐dose GM‐CSF with optimized medium and prolonged differentiation. Flt3L, Flt3L+SCF, iCD103, and iDC1 were compared directly under standardized conditions [14], whereas Flt3L+IL‐4 was evaluated separately [29]; quantitative comparisons involving the IL‐4 platform are therefore indirect.

## 4 Notch Ligand‐Supported cDC1 Generation

The established role of Notch2 in the differentiation and homeostasis of selected cDC subsets in vivo, most prominently in specific cDC2 populations, prompted efforts to incorporate membrane‐bound developmental signals into in vitro cDC1‐generation systems [35, 36, 37]. This led to the development of stromal‐supported co‐cultures using OP9 cells expressing the Notch ligands Delta‐like 1 (DLL1) or Delta‐like 4 (DLL4) together with Flt3L (Figure 1B) [13, 38]. These systems generate mixed cDC cultures containing both cDC1s and cDC2s. In murine cultures, Notch ligand exposure promotes expression of CD8α, CD103, and DEC205, shifts cDC1 transcriptional profiles toward those of primary splenic cDC1s, and enhances C–C motif chemokine receptor 7 (CCR7) expression, migration toward CCR7 ligands, CD8^+^ T‐cell cross‐priming, and antitumor vaccination efficacy [13].

Terminology for cytokine‐generated murine cDC1s has varied with the benchmark applied. Naik et al. described Flt3L‐derived CD24^hi^ cells as bona fide counterparts of splenic CD8α+ DCs based on developmental, phenotypic, and functional evidence [24], whereas Kirkling et al. later termed Flt3L‐derived cells immature “cDC1‐like” and described iCD103‐DC cDC1s as sharing an “aberrant” surface phenotype relative to primary splenic cDC1s [13]. A subsequent standardized comparison showed distinct culture composition, cDC1 purity, and yield across Flt3L, Flt3L+SCF, iCD103, and iDC1 systems, while RNA sequencing and ImmGen analysis aligned sorted Flt3L‐, iCD103‐, and iDC1‐derived cDC1s with the cDC1 lineage [14]. These data support classification of the analyzed populations as bona fide cDC1s with culture‐dependent differentiation states [14, 19, 24]. In primary murine bone marrow cultures, Notch signaling therefore promotes acquisition of splenic cDC1‐associated phenotype and function rather than establishing cDC1 identity de novo. Notch‐supported cultures complement the directly compared Flt3L, Flt3L+SCF, iCD103, and iDC1 systems by providing a membrane‐bound differentiation signal, but acquisition of CD8α, CD103, or related markers does not itself establish equivalence to a specific tissue cDC1 state.

Available human progenitor studies suggest a substantially larger quantitative effect of Notch ligand exposure on cDC1 output. DLL1‐supported cultures markedly increased generation of IRF8‐dependent CD141^+^ cDC1s from human bone marrow progenitors [13]. Independent human CD34^+^ progenitor systems similarly identified cooperation between Notch and GM‐CSF and demonstrated phenotypic, functional, and single‐cell transcriptional similarity to blood cDC1s [39]. These data support cDC1 lineage assignment, but the cultures also contain cDC2s, pDCs, and other populations; Notch therefore increases cDC1 output without being cDC1‐selective [39]. Whether the larger effect in human systems reflects an intrinsic species difference or differences in progenitor source, cytokine composition, ligand density, feeder support, culture duration, or experimental endpoints remains unclear. No specific compensatory pathway explaining murine cDC1 generation without experimentally supplied Notch ligands has been established.

The juxtacrine nature of Notch signaling raises the question of whether comparable ligand‐restricted niches operate in vivo. In the human thymus, a spatially restricted JAG1^+^ medullary niche supports GATA2‐dependent generation of progenitors with cDC, pDC, and monocyte potential from early thymic progenitors [40]. DLL1 suppresses this myeloid program at the early progenitor stage, whereas both DLL1 and JAG1 support survival and DC development after common DC progenitor (CDP) formation [40]. Thus, Notch effects depend on ligand and developmental stage. These findings establish a localized permissive environment for human DC‐lineage development, but not a universal or cDC1‐specific Notch requirement, nor do they establish a functional role for NOTCH4. Whether analogous niches regulate cDC1 development or abundance in other human lymphoid organs or the liver remains unknown.

In mice, steady‐state Notch2 requirements are more firmly established for selected cDC2 populations than for general cDC1 specification [35, 36, 37]. Nevertheless, DC‐intrinsic Notch2 deficiency in a model of inflammation‐associated colorectal cancer impaired terminal cDC differentiation, reduced CCR7 expression and migration, and suppressed antigen cross‐presentation, supporting a context‐dependent role for Notch2 in terminal DC differentiation and cDC1‐associated antitumor immunity [41].

Important mechanistic questions remain unresolved. Murine culture studies implicate NOTCH2 [13], whereas the receptor or receptor combination responsible for human cDC1 generation has not been functionally established; NOTCH4 expression in human thymic DC‐lineage cells is suggestive but does not demonstrate a requirement [40]. Human CD34^+^ studies indicate early Notch activity [39], whereas murine culture and disease models support later roles in terminal differentiation and function [13, 41]. The responding progenitor, duration of signaling, and requirement at multiple stages remain unclear. Although Notch and GM‐CSF cooperate, their molecular integration with Flt3L and other feeder‐derived signals is undefined [13, 39]. Transcriptomic changes have been described [13, 39], but direct Notch targets, effects on lineage‐defining transcription factors, and chromatin accessibility remain incompletely characterized.

From a practical perspective, feeder dependence, multilineage output, inconsistent whole‐culture reporting, and limited head‐to‐head comparisons reduce standardization and constrain evaluation of cDC1 selectivity. Macrophage‐like cells, residual progenitors, and other non‐DC populations are not uniformly quantified, making cDC1 purity and absolute yield difficult to compare with cytokine‐driven platforms [13, 39]. Disease‐associated changes in Notch‐ligand expression or therapeutic manipulation of the pathway could also alter DC differentiation, migration, or function, although effects are likely to depend on ligand, receptor, tissue, and developmental stage, and direct clinical evidence for cDC1‐specific consequences remains limited. Overall, Notch‐supported cultures show that membrane‐bound cues can substantially alter cDC1 output, differentiation state, and function while underscoring the need for defined feeder‐free systems, standardized whole‐culture reporting, and greater mechanistic resolution.

## 5 Human cDC1 Generation From CD34+ Progenitors

The rarity of circulating human cDC1s has driven extensive efforts to generate these cells ex vivo from CD34^+^ hematopoietic progenitors (Figure 1C). Early Flt3L‐based cultures generated multiple human DC subsets, including CD141^+^CLEC9A^+^ cDC1s, CD1c^+^ cDC2s, and CD123^+^ pDCs [15, 42, 43]. Within these heterogeneous cultures, XCR1^+^CD141^+^CLEC9A^+^ cells showed phenotypic, transcriptional, and functional similarity to blood cDC1s, supporting assignment to the cDC1 lineage [15]. However, cDC1s remained a minority of the total output, and these systems were not optimized for selective, high‐purity cDC1 production.

Subsequent approaches sought to increase cDC1 output and better reproduce developmental niche signals. Notch ligand‐expressing feeder systems markedly increased cDC1 generation but remained multilineage, whereas engineered mesenchymal stromal niches supported broader human DC development through membrane‐bound niche factors [13, 39, 44]. These studies established the importance of cell‐contact‐dependent signals but did not provide selective, feeder‐free cDC1 production.

More recent efforts have focused on improving scalability and translational applicability. Addition of IL‐4 to Flt3L cultures of human CD34^+^ progenitors likewise markedly increased CLEC9A^+^CD141^+^ cDC1 output, although complete whole‐culture composition was not reported [29]. A feeder‐free Flt3L and GM‐CSF protocol generated large numbers of CD141^+^CLEC9A^+^ cells, but because comprehensive developmental and transcriptional benchmarking was not reported, these products remain best described as cDC1‐like under the definitions used here [45]. A serum‐free DLL1‐supported system generated large‐scale cDC1 output and benchmarked the resulting cells against blood cDC1s by phenotype, transcriptomics, cross‐presentation, and preclinical vaccination, supporting cDC1 lineage assignment; however, the reported high‐output configuration remained feeder‐dependent and preclinical [46]. In a recent preprint, a good manufacturing practice (GMP)‐compatible feeder‐free workflow combining cytokine optimization with StemRegenin 1‐mediated progenitor expansion reported high cDC1 yields and phenotypic and single‐cell transcriptional similarity to primary human cDC1s [47].

Collectively, human CD34^+^ platforms span heterogeneous cytokine cultures, feeder‐dependent niche systems, and emerging feeder‐free workflows. Direct comparisons remain limited because starting material, culture duration, yield denominators, lineage benchmarking, and reporting of monocyte/macrophage‐like cells, residual progenitors, and other non‐DC populations vary among studies. Scalable or GMP‐compatible design should therefore not be equated with demonstrated clinical readiness.

## 6 iPSC‐Derived cDC1‐Like Cells

iPSCs represent an alternative, renewable source for generating human DCs (Figure 1D). Early studies demonstrated that iPSCs could be differentiated into heterogeneous cultures containing CD141^+^XCR1^+^ DCs with cDC1‐associated phenotypic and functional properties, including cross‐presentation, establishing proof‐of‐principle for generating human cDC1‐like cells from renewable stem‐cell sources [48, 49]. However, these cells were not comprehensively benchmarked developmentally or transcriptionally against primary cDC1s, and one protocol reported fetal and tolerogenic features that distinguished them from adult blood cDC1s [48].

Subsequent work showed that DLL1‐mediated Notch signaling promotes differentiation of iPSC‐derived CD34^+^ progenitors toward cDC lineages, including CD141^+^XCR1^+^CLEC9A^+^ cells with single‐cell transcriptional similarity to blood cDC1s [50]. The cultures nevertheless remained heterogeneous and also contained cDC2, DC3, and other DC populations [50]. More recent feeder‐free expansion of iPSC‐derived hematopoietic progenitors increased DC output, but the resulting phenotypically defined cDC1‐like, cDC2, and DC3 populations were transcriptionally most similar to monocyte‐derived DCs rather than primary cDC subsets [51].

Thus, iPSC‐derived systems provide a renewable and engineerable source of human cDC1‐like cells, but lineage correspondence remains protocol‐dependent. Heterogeneous output, prolonged multistep differentiation, and incomplete correspondence to primary cDC1s remain important limitations, and clinical readiness has not been established [50, 51].

## 7 Transcription Factor‐Based Programming

Direct lineage reprogramming has recently emerged as an alternative to stem‐cell‐based differentiation by converting somatic cells directly into DCs through enforced expression of lineage‐defining transcription factors (Figure 1E). Rosa et al. identified the combination of PU.1, IRF8, and BATF3 as sufficient to reprogram mouse and human fibroblasts into induced DCs with a cDC1‐like transcriptional program and antigen‐presenting functions; murine induced DCs also cross‐presented antigen to CD8^+^ T cells [52].

Subsequent studies used single‐cell transcriptomics to define successful reprogramming trajectories and identify factors limiting human cDC1 generation. Human induced DCs progressively acquired a global cDC1 transcriptional profile without induction of other DC subsets, and addition of interferon gamma, interferon beta, and tumor necrosis factor alpha to optimized PU.1, IRF8, and BATF3 expression markedly increased reprogramming efficiency while enabling generation from adult fibroblasts and mesenchymal stromal cells [53]. Mechanistically, PU.1 engaged chromatin early and recruited IRF8 and BATF3 to shared regulatory sites, activating cDC1‐associated programs while suppressing fibroblast identity [53].

Adenoviral delivery of PU.1, IRF8, and BATF3 within established tumors reprogrammed tumor cells into cDC1‐like antigen‐presenting cells, promoting tertiary lymphoid structure formation, expansion of polyclonal T‐cell responses, and durable antitumor immunity in multiple melanoma models [54].

Together, these studies establish direct transcription factor‐mediated programming as a distinct strategy for inducing cDC1‐like states without recapitulating progenitor differentiation. The generated cells show substantial phenotypic, transcriptional, and functional correspondence to cDC1s, but reprogramming efficiency and completeness vary. Under the terminology used here, they remain best described as cDC1‐like cells because normal cDC1 ontogeny is bypassed and equivalence to primary cDC1 states remains incompletely established. These approaches remain preclinical, with delivery specificity, safety, and stability of the reprogrammed state requiring further evaluation.

## 8 Expansion and Mobilization of Endogenous cDC1s

An alternative to ex vivo cDC1 generation is the expansion and mobilization of endogenous cDC1s in vivo (Figure 1F). Early studies demonstrated that systemic Flt3L administration markedly expands DCs in mice and humans, establishing proof‐of‐principle that endogenous cDC populations can be therapeutically manipulated [20, 21]. Flt3L expands several Flt3‐responsive DC populations rather than acting selectively on cDC1s, and increased cDC1 abundance should therefore be distinguished from direct evidence that cDC1s mediate therapeutic efficacy [20, 21, 55].

Flt3L‐mediated expansion alone may be insufficient to control established tumors. Accordingly, Flt3L is commonly combined with radiotherapy or other sources of antigen release, Toll‐like receptor (TLR) agonists or CD40 agonists, immune checkpoint blockade, and antigen‐directed vaccination [6, 12, 55, 56, 57]. In the in situ immunomodulation regimen developed by Oba et al., Flt3L increased tumor‐resident cDC1s, but robust tumor regression required radiotherapy together with TLR3 and CD40 stimulation [57]. Therapeutic efficacy fell to the level of radiotherapy alone in wild‐type hosts reconstituted with Batf3‐deficient bone marrow, demonstrating dependence on Batf3‐dependent bone marrow‐derived cells and providing strong mechanistic evidence for cDC1 involvement [57]. These findings show that cDC1 expansion can be mechanistically important in defined regimens but must be coordinated with antigen availability, activation, and migration; the extent of cDC1 dependence remains treatment‐ and tumor‐context‐specific [6, 55].

Other approaches seek to improve cDC1 expansion or function. Engineering Flt3L as an albumin fusion protein prolonged serum half‐life and enhanced accumulation within tumors and draining lymph nodes, resulting in greater intratumoral cDC1 expansion than recombinant Flt3L [56]. In a distinct transient strategy, lipid nanoparticle delivery of IRF8‐ or NF‐κB‐inducing kinase‐encoding messenger RNA activated antigen‐presenting cells, increased activated cDC1s in tumors and tumor‐draining lymph nodes, enhanced antitumor CD8^+^ T‐cell responses, and produced durable therapeutic activity; co‐delivery with antigen‐encoding messenger RNA further increased antigen‐specific immunity [58]. This approach remodels endogenous antigen‐presenting‐cell states rather than directly reprogramming somatic cells into cDC1‐like cells.

Collectively, endogenous expansion and mobilization complement ex vivo generation. These approaches preserve physiological antigen acquisition and tissue localization but provide less control over cell identity, dose, and activation state and can affect multiple Flt3‐responsive populations. Their therapeutic activity therefore depends not only on increasing cDC1 abundance but also on coordinating antigen release, activation, trafficking, and relief of local immunosuppression.

## 9 Choosing the Appropriate cDC1 Strategy

No single approach is optimal for all applications, and the choice of cDC1 strategy should be guided by the biological question and intended application (Table 1). Relevant criteria include species and starting material, strength of lineage validation, whole‐culture composition, cDC1 purity and absolute output, culture duration, genetic tractability, and translational maturity. Conventional Flt3L and Flt3L+SCF bone marrow cultures are well‐suited to mechanistic studies of DC development and progenitor biology, whereas iCD103 and iDC1 are preferable when enriched or high‐purity murine cDC1s are required, at the cost of longer culture. Notch‐supported systems enable analysis of membrane‐bound niche signals but remain feeder‐dependent, multilineage, and incompletely standardized. Human CD34^+^ progenitor platforms range from heterogeneous developmental models to scalable cDC1‐production systems and should be selected according to lineage benchmarking, feeder dependence, and manufacturing requirements. iPSC‐derived approaches provide renewable and engineerable sources of cDC1‐like cells but require prolonged multistep differentiation and remain protocol‐dependent. Direct transcription factor‐mediated programming bypasses progenitor stages and can rapidly induce cDC1‐like states, but remains preclinical and requires further evaluation of delivery specificity, safety, and stability.

The choice between ex vivo generation and endogenous expansion or mobilization also depends on the therapeutic context. Ex vivo approaches can provide greater control over cell composition, antigen loading, activation, engineering, and release testing, but only when identity, purity, and potency are rigorously established. Endogenous strategies preserve tissue context and physiological antigen acquisition but affect multiple Flt3‐responsive populations and provide less control over dose and activation state; therapeutic activity therefore generally requires coordinated antigen release, activation, trafficking, and relief of local immunosuppression. Scalable or GMP‐compatible design should not be equated with demonstrated clinical readiness. Standardized benchmarking against relevant primary cDC1 states and direct comparisons between platforms will be essential to match each approach to the biological or translational question it is best suited to address.

## 10 Conclusions and Future Perspectives

Over the past two decades, strategies for generating and expanding cDC1s have evolved from heterogeneous cytokine cultures into a diverse toolkit encompassing optimized cytokine‐driven differentiation, stromal‐supported systems, human CD34^+^ progenitor cultures, iPSC‐derived cDC1‐like cells, transcription factor‐mediated programming, and endogenous cDC1 expansion (Figure 1). These approaches generate products ranging from heterogeneous or cDC1‐enriched cultures to high‐purity bona fide cDC1s and cDC1‐like cells and should therefore be considered complementary rather than interchangeable.

Despite this progress, important challenges remain. No single marker or function is sufficient to establish cDC1 identity, and comparisons among platforms require convergent developmental, phenotypic, transcriptional, and functional evidence. Studies should report starting material, culture duration, cell density, media and cytokine conditions, complete cellular composition using a common denominator, absolute cDC1 output, and the primary cDC1 reference populations used for benchmarking. Complete whole‐culture reporting is particularly needed for Flt3L+IL‐4, Notch‐supported, and human progenitor systems, in which macrophage‐like cells, residual progenitors, and other non‐DC populations are not uniformly quantified. Greater standardization and systematic head‐to‐head comparisons will be essential for both mechanistic studies and translational development.

Current platforms address distinct biological and technical bottlenecks rather than forming a universal hierarchy (Figure 2). The evolution of mouse cytokine systems illustrates how progenitor expansion, lineage differentiation, cDC1 survival and maintenance, media composition, and culture kinetics can be combined rationally, but quantitative superiority should be inferred only from direct standardized comparisons. Future work should define the receptor usage, developmental windows, cytokine cooperation, and downstream transcriptional and chromatin programs through which Notch regulates mouse and human cDC1 differentiation. Additional priorities include developing feeder‐free and compositionally defined human systems and establishing reproducible criteria for identity, potency, manufacturing consistency, and clinical readiness.

Ex vivo generation and endogenous expansion remain complementary strategies. Ex vivo platforms offer greater control over cell composition and experimental manipulation, whereas endogenous approaches preserve tissue context and physiological antigen acquisition. Matching these distinct capabilities to specific mechanistic and translational applications, while rigorously validating lineage identity and function, will determine the utility of each platform.

## Author Contributions

S.S. (Writing – original draft, Writing – review and editing); S.P. (Writing – original draft, Writing – review and editing); C.T.M. (Writing – original draft, Writing – review and editing, funding acquisition, supervision).

## Conflicts of Interest

The authors declare no conflicts of interest.

## Permission to Reproduce Material from Other Sources

Figures 1 and 2 were created by the authors using BioRender.com under publication licenses. No material was reproduced or adapted from previously published sources.

## Data Availability

Data sharing not applicable to this article as no datasets were generated or analysed during the current study.
